# Vitamin D_3_ Supplementation Reduces the Symptoms of Upper Respiratory Tract Infection during Winter Training in Vitamin D-Insufficient Taekwondo Athletes: A Randomized Controlled Trial

**DOI:** 10.3390/ijerph15092003

**Published:** 2018-09-14

**Authors:** Hyun Chul Jung, Myong-Won Seo, Sukho Lee, Sung Woo Kim, Jong Kook Song

**Affiliations:** 1 Department of Kinesiology, College of Health Sciences, University of Louisiana at Monroe, 700 University Avenue, Monroe, LA 71209, USA; jung@ulm.edu; 2 Department of Taekwondo, College of Physical Education, Kyung Hee University, 1732 Deokyoungdaero, Giheung-gu, Yongin-si, Gyeonggi-do 17014, Korea; smilly1004@khu.ac.kr (M.-W.S.); kswrha@khu.ac.kr (S.W.K.); 3 Department of Counseling, Health, and Kinesiology, College of Education and Human Development, Texas A&M University-San Antonio, One University Way, San Antonio, TX 78224, USA; slee@tamusa.edu

**Keywords:** high-intensity training, respiratory immune function, secretory immunoglobulin, vitamin D

## Abstract

Vitamin D insufficiency may be associated with increased risk of upper respiratory tract infection (URTI) in athletes. This study examined the effects of vitamin D_3_ supplementation on salivary immune functions and symptoms of URTI in vitamin D-insufficient taekwondo athletes. Twenty-five male taekwondo athletes, aged 19–22 years with vitamin D insufficiency [serum 25-hydroxyvitamin-D concentrations (25(OH)D, 31.3 ± 1.39 nmol/L)], participated in this study. They were randomized to receive 5000 IU/day of vitamin D_3_ (*n* = 13) or placebo capsule (*n* = 12) during 4 weeks of winter training. Blood samples were collected two times (pre- and post-tests) for analyzing serum 25(OH)D concentration while salivary samples were obtained three times (pre-, mid-, and post-tests) for secretory immunoglobulin A (SIgA) and lactoferrin analyses. The symptoms of URTI were reported daily during the intervention. Serum 25(OH)D concentration significantly increased by 255.6% in the vitamin D group, whereas in the placebo group it did not change (*p* < 0.001). While the significant increase in SIgA was observed in both groups (*p* < 0.001), elevated salivary lactoferrin level in response to winter training was found only in the placebo group (*p* = 0.011). The change in serum 25(OH)D concentration was negatively associated with total URTI symptoms (*r* = −0.435, *p* = 0.015). Vitamin D_3_ supplementation may be effective in reducing the symptoms of URTI during winter training in vitamin D-insufficient taekwondo athletes.

## 1. Introduction

Athletes of many sports participate in various training programs such as high-intensity interval training, strength training, and conditioning training to improve their athletic performance [1,2]. Although high-intensity training provides performance benefits, there is a physiological challenge to immune functions [3,4]. Traditionally, it has been recognized that participating in moderate-intensity exercise comes with a lower risk of illness, whereas engaging in excessive volume of high-intensity exercise suppresses the immune functions, thus the risk of illness is increased. This concept has been introduced by Nieman known as ‘J-Curve’ [5]. The author pointed out that the intensity and volume of exercise play important roles in modulating the immune functions.

Upper respiratory tract infection (URTI) is the most common non-injury-related illness among sports athletes, accounting for a 35%–65% incidence rate during high-intensity training or in competition [6,7,8]. The acute URTI has been observed following heavy exercise in professional soccer players [9], and high ventilation rates during high-intensity training has been known to contribute to the exposure to bacterial and viral pathogens from the environment [10]. Additionally, prolonged overtraining has shown to decrease respiratory immune functions [11]. It has been demonstrated that the incidence of URTI was associated with reduced training load by 24% in endurance sports athletes [12], and this insufficient training load may increase the injury rates and decrease the physical capacity [13,14]. Besides, inadequate nutrition status during heavy exercise can impair the respiratory immune functions [15]. Therefore, a well-designed training program with an adequate nutritional supplementation is needed, not only to prevent the respiratory infections but also to optimize the performance [16].

Recently, vitamin D_3_ supplementation has received wide attention in the athletic communities because vitamin D deficiency or insufficiency is a prevalent issue among athletes such as swimmers [17], football athletes [18], wrestlers [19], and taekwondo athletes [20]. Vitamin D deficiency has been associated with low physical performance level as well as high susceptibility to URTI. There was a negative linear association between vitamin D status and URTI in British adults [21], and vitamin D-deficient athletes were more likely to present URTI symptoms than optimal vitamin D groups [12]. It has been recognized that vitamin D plays a vital role in modulating innate immune functions. Liu and colleagues reported that the activation of toll-like receptors was linked with vitamin D-mediated antimicrobial protein (AMP) production [22], and the concentration of AMPs, including secretory immunoglobulin A (SIgA) and plasma cathelicidin, was positively associated with vitamin D status in endurance sports athletes [12]. However, it is still debatable whether correcting vitamin D insufficiency reduces URTI and improves salivary immune functions. Recently, meta-analysis studies have found that vitamin D supplementation prevents the acute URTI [23,24], but the subjects in the selected studies were primarily children or patients. We believe that lack of studies with sports athletes may limit confirmation of the efficacy of vitamin D supplementation on URTI in the athletic communities. Previous studies have found that while 14 weeks of vitamin D_3_ supplementation improve the resistance to respiratory infections in athletes [25], adolescent swimmers did not reduce URTI following 12 weeks of vitamin D_3_ supplementation [17].

Taekwondo athletes may be more likely to present low vitamin D levels, since the majority of their training takes place indoors, therefore decreasing their sunlight exposure time. Also, the weight category in sport may decrease the athletes’ vitamin D supplementation via food intake [20]. Taekwondo athletes generally prepare for their upcoming season during the winter period and training programs include various types of high-intensity interval training and endurance training [2,26]. We believe that vitamin D insufficiency during winter training may negatively influence the athletes’ respiratory immune functions. Therefore, this study examined the effects of vitamin D_3_ supplementation on salivary immune functions and URTI symptoms during winter training in vitamin D-insufficient taekwondo athletes. It was hypothesized that four weeks of vitamin D_3_ supplementation will increase serum 25(OH)D level. An increased serum 25(OH)D level will improve salivary immune functions and symptoms of URTI in vitamin D-insufficient taekwondo athletes.

## 2. Methods

### 2.1. Participants

Twenty-six collegiate male taekwondo athletes, aged 19–22 years with vitamin D insufficiency (<50 nmol/L), were initially enrolled in the study. Subjects regularly participated in both physical and technical training five times a week, at least three hours per day. Before the study, subjects were primarily screened with a questionnaire about their health status. The inclusion criteria of the study were as follows: (a) healthy subject, (b) no skeletomuscular injuries, (c) no URTI episodes at least one week before the study, and (d) lower than 50 nmol/L of serum 25(OH)D concentration at baseline. Any athletes who (a) were not willing to participate, (b) were taking any vitamin D_3_ supplement within the last six months, and (c) had greater than 50 nmol/L of serum 25(OH)D concentration at baseline were excluded. After screening of inclusion and exclusion criteria, eligible subjects were randomly allocated to the vitamin D group (*n* = 13) and the placebo group (*n* = 13). However, one subject from the placebo group could not complete the study due to personal reasons, therefore 25 male taekwondo athletes (age: 19.9 ± 1.85 years, career: 99.8 ± 4.49 months, height: 181.7 ± 1.17 cm) completed this study. Each subject signed a written consent form that had been approved by the Institutional Review Board of the University (KHSIRB-15-025).

### 2.2. Study Design

**Figure 1:**
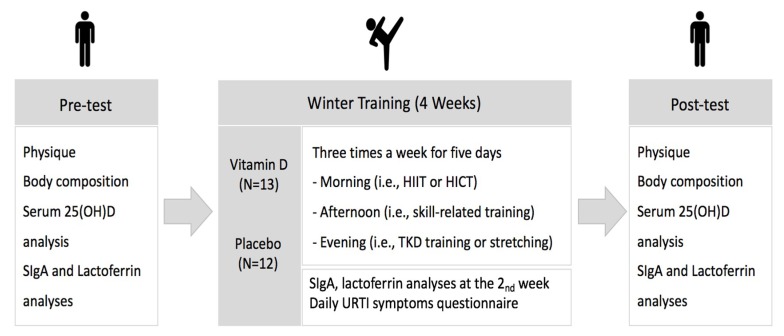
Study procedure note. HIIT: high intensity interval training, HICT: high intensity continuous training, SIgA: secretory immunoglobulin A, URTI: upper respiratory tract infection.

This study was designed to be randomized, double-blinded, and placebo-controlled. The block randomization was applied based on serum 25(OH)D concentration. Participants’ serum 25(OH)D concentrations were analyzed at baseline to examine the vitamin D status. In this study, vitamin D insufficiency was defined as serum 25(OH)D level of 30–50 nmol/L [27]. All taekwondo athletes were vitamin D-insufficient at baseline (31.3 ± 1.39 nmol/L). Eligible subjects then were randomly allocated to the vitamin D and placebo groups using block design with blocks of four or two to keep the group sizes equal. They received daily either vitamin D_3_ (5000 IU) or a visually identical placebo capsule (Bio-Tech Pharmacal Inc., Fayettville, AR, USA) during four weeks of winter training. The treatment was managed in a double-blinded fashion where subjects, coach, and investigators remained unaware of the actual product during the intervention. Briefly, one evaluator who was not directly involved in measurements and training provided either vitamin D_3_ or placebo capsules to subjects. The pre- and post-tests were performed three days before and after the intervention. Blood samples were collected two times (pre- and post-tests) to analyze serum 25(OH)D concentration while salivary samples were obtained three times (pre-, mid-, and post-tests) for SIgA and lactoferrin analyses. The symptoms of URTI were daily reported by questionnaire during the intervention (Figure 1).

### 2.3. Physique and Body Composition

Subjects’ height and body weight were measured to the nearest 0.1 cm and 0.1 kg, respectively, by using a stadiometer (Takei, T.K.K., Japan) and a calibrated scale (Seca 700, Seca GmbH, Hamburg., Germany). Body composition was assessed by dual X-ray absorptiometry (DXA, QDR-4500W, Hologic, Marlborough, MA, USA). Subjects wore light clothes without any metal and removed the jewelry attached to their body. Then, subjects were asked to lie on the DXA table in the supine position without any movement. A whole-body DXA scan was performed for seven minutes. The DXA analysis was performed by the same-trained technician. The intraclass correlation coefficients (ICC) of DXA measurement was 0.99 in our laboratory [28]. Lean body mass (kg), fat mass (kg), and body fat percentage (%) were reported in this study.

### 2.4. Winter Training

Four weeks of winter training were conducted on the Jeju island in South Korea (latitude 33°29′ N). The training program was designed based on previous studies, which included stretching, strength, and conditioning, and technical training [2,26]. Briefly, subjects participated in three sessions of training a day for five days a week. In the morning session (9:30 a.m.–11:30 am), subjects performed high-intensity continuous running (>85% HR_max_) or high-intensity intermittent running (>90% HR_max_) on Monday, Wednesday, and Friday while they did resistance training on Tuesday and Thursday. In the afternoon session (3:00 p.m.–5:00 p.m.), subjects performed various skill-related training such as ladder drills and plyometric training. In the evening session (8:00 p.m.–9:00 p.m.), subjects performed taekwondo training (i.e., basic kicking, sparring) or stretching. All subjects were asked to report their rating of perceived exertion (RPE) with Borg’s scale (i.e., ‘6’ no exertion at all, ‘13’ somewhat hard, ‘20’ maximal exertion) at the end of the day to monitor daily perceived training intensity. All subjects stayed in the same dormitory and a registered dietitian provided the meals during intervention.

### 2.5. Serum 25OH(D) Concentration Analysis

Blood samples were collected two times pre- and post-tests to analyze serum 25(OH)D concentration. Blood sample collection was performed in the morning between 8:00 a.m. and 9:00 a.m. Subjects were asked to overnight fast and were prohibited from any severe physical activity for 24 h before the test. When subjects arrived in the laboratory, they sat on the chair quietly for 10 min. Then, venous blood samples (3 mL) were obtained from the antecubital vein by a certified technician. The collected blood samples were clotted for ten minutes at room temperature (20–22 °C) and centrifuged at 3000 rpm for 15 min. The separated serum samples were stored at 80 °C. An automatic chemiluminescent immunoassay (CLIA) analyzer (Liaison XL, Diasorin, Saluggia, Italy) with Serum 25(OH)D kit (Liaison 25 OH vitamin Total, Diasorin, Saluggia, Italy) were used to analyze serum 25(OH)D concentration. The variations of serum 25(OH)D analysis were less than 5%. The laboratory participates in the International External Quality Assessment Scheme for Vitamin D metabolites (DEQAS).

### 2.6. SIgA and Lactoferrin Analysis

Salivary samples were obtained three times in the morning between 8:00 a.m. and 9:00 a.m. at pre-, mid- (2nd week), and post-tests to analyze the SIgA and lactoferrin concentrations. Subjects were instructed not to drink any alcohol for 12 h with at least 3 h of fasting before the sample collection. Subjects were prohibited from brushing their teeth one hour before the sample collection to prevent any blood contamination. Prior to sample collection, subjects rinsed their mouth for 10 min with cool water. Subjects then tilted their head slightly forward and provided salivary samples in the conical tube (3 mL). While collecting the salivary samples, subjects were asked to minimize orofacial movement. The collected samples were stored at −80 °C. Enzyme-Linked Immunosorbent Assay (ELISA) methods were used for analyzing SIgA and lactoferrin concentrations with salivary secretory IgA indirect enzyme immunoassay kit (Salimetrics, Carlsbad, CA, USA) and human lactoferrine ELISA kit (Biovendor, Brno, Czech Republic) by a microplate reader (VERSA Max Molecular device, San Jose, CA, USA). The variations of SIgA and lactoferrin assays were less than 5%. Both blood and salivary samples were analyzed at the certified research laboratory (Green cross lab cell, Yongin, Korea).

### 2.7. Upper Respiratory Tract Infection Symptoms 

The symptoms of URTI were reported daily during four weeks of winter training. The Wisconsin Upper Respiratory Symptom Survey-11 (WURSS-11) was used in this study. The questionnaire has been validated in a previous study [29]. Briefly, the questionnaire included one overall URTI question, six URTI symptom questions, three quality of life (QOL) questions, and one overall URTI change question. The URTI symptom represented the condition of nasal and throat, while QOL indicated the condition of daily activities, thinking, and tiredness. The severity of URTI symptoms was rated on a 7-point Likert scale: 1 (very mild), 3 (mild), 5 (moderate), and 7 (severe). The URTI symptom score was calculated by summing of six URTI symptom questions while the QOL score was calculated by summing of three QOL questions during the study. The total URTI symptom is an overall condition expressed by summing of URTI symptom and QOL scores [26]. In the present study, the average score (sum of total score divided by the number of collecting day, score/day) of each variable was reported. During the intervention period, eight subjects did not complete the questionnaires, so 17 questionnaires (vitamin D, *n* = 8; placebo, *n* = 9) were used in the present study.

### 2.8. Statistical Analysis

Statistical analysis was performed by the SPSS software program (version 25, SPSS Inc., Chicago, IL, USA). First, independent *t*-test was applied to examine the difference of baseline data between the vitamin D and the placebo groups. Repeated measures of analysis of variances (ANOVAs) were performed for all parametric variables to examine the interaction effects for group by time. If any significant interaction or main effects were observed, paired *t*-test for 25(OH)D concentration or Bonferroni post hoc test for salivary variables was performed. Mann-Whitney *U* test was applied for nonparametric variables (i.e., URTI symptom, QOL, total URTI symptom) between the vitamin D and the placebo groups. Kendall’s rank order correlation test was used to examine the correlations between the absolute change in serum 25(OH)D concentration and total URTI symptom. All of the data was presented by mean and standard error. Effect sizes (ESs) were reported by partial eta squared (*η*_p_^2^) [30]. The significance level was set at 0.05 in this study.

## 3. Results

### 3.1. Changes in Body Composition Variables

Table 1 presents the changes of body composition variables during winter training. There were no significant differences in body composition variables between the vitamin D and the placebo groups at baseline (*p* > 0.05). No significant interaction effects for group by time on body composition variables were observed. However, there were significant time effects on body weight (*p* < 0.001, *η*_p_^2^ = 0.486), fat mass (*p* = 0.015, *η*_p_^2^ = 0.023), and body fat percentage (*p* < 0.001, *η*_p_^2^ = 0.634) where both groups decreased significantly from pre- to post-tests.

### 3.2. Change in Serum 25(OH)D Concentration

**Figure 2:**
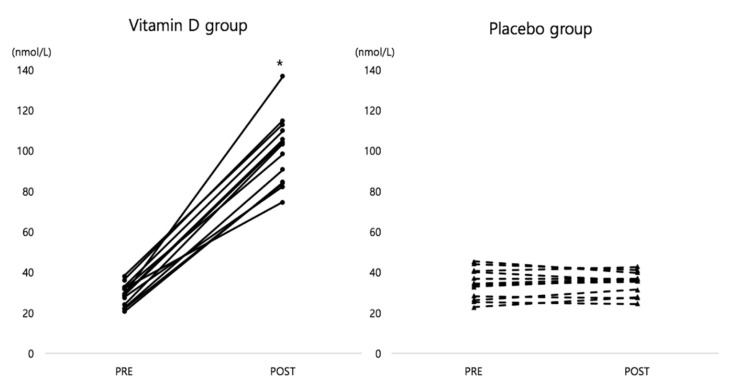
Individual changes of serum 25(OH)D concentration from pre- to post-tests. * *p* < 0.05 indicates a significant increase from pre- to post-tests in the vitamin D group.

There was a significant interaction effect for group by time on serum 25(OH)D concentration (*F* = 253.316, *p* < 0.001, *η*_p_^2^ = .917). While the vitamin D group significantly increased serum 25(OH)D concentration from 28.7 ± 1.51 to 100.1 ± 4.70 nmol/L, in the placebo group, it did not change significantly (PRE: 34.2 ± 2.16 and POST: 34.6 ± 1.66 nmol/L) during winter training. Figure 2 shows the individual changes of serum 25(OH)D concentration from pre- to post-tests.

### 3.3. Changes in Salivary Immune Functions

**Figure 3:**
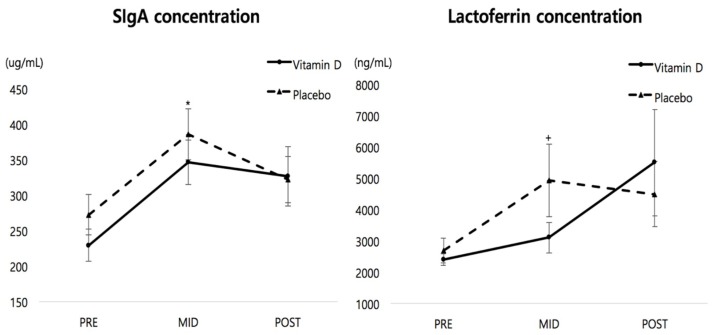
Changes in SIgA and lactoferrin concentrations. Note: * *p* < 0.05 indicates that SIgA concentration increased in both groups from pre- to mid -tests, ^+^ *p* < 0.05 indicates that lactoferrin concentration increased only in the placebo group from pre- to mid-tests.

Figure 3 shows the changes of SIgA and lactoferrin concentrations. There were no significant interaction effects for group by time on SIgA and lactoferrin concentrations (*p* > 0.05). However, there were significant time effects on SIgA (*F* = 23.0, *p* < 0.001, *η*_p_^2^ = 0.511) and lactoferrin (*F* = 5.788, *p* = 0.011, *η*_p_^2^ = 0.208). Bonferroni post hoc tests showed that while the significant increase in SIgA concentration was observed in both groups, the elevated salivary lactoferrin level in response to winter training was found only in the placebo group.

### 3.4. Changes in URTI Symptoms

**Figure 4:**
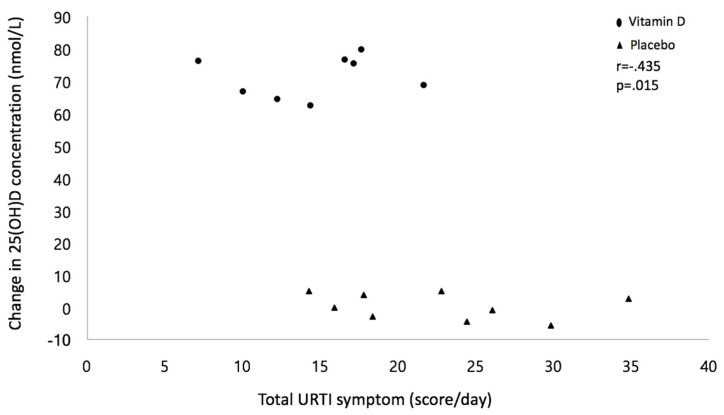
Correlation between the change in 25(OH)D concentration and total URTI symptom. Note: number of missing data from the vitamin D group was five while the placebo group was three; the correlations within each group were not significant (Vitamin D group, *r* = 0.143, *p* = 0.621, Placebo group, *r* = −0.366, *p* = 0.173, respectively).

Table 2 presents the difference of URTI symptoms between the vitamin D and placebo groups. There were significant differences in URTI symptoms (i.e., runny nose, sneezing, cough) and total URTI symptoms between the groups where the vitamin D group was lower than the placebo group. However, the quality of life score (i.e., tiredness, daily activity) was not different between groups. The change in serum 25(OH)D concentration was negatively associated with total URTI symptoms during winter training. However, the significant correlations within each group were not observed in the study (Figure 4).

## 4. Discussion

The aim of this study was to examine the effects of vitamin D_3_ supplementation on salivary immune functions and URTI symptoms in vitamin D-insufficient taekwondo athletes. Major findings of the study were that (1) daily dose of 5000 IU of vitamin D_3_ increased serum 25(OH)D level to a sufficient level, (2) while the increase in SIgA concentration was observed in both groups, the elevated salivary lactoferrin concentration in response to winter training was found only in the placebo group, and (3) the change in serum 25(OH)D concentration was negatively associated with total URTI symptoms during winter training in vitamin D-insufficient taekwondo athletes.

### 4.1. Vitamin D Supplementation and Salivary Immune Functions

Monitoring the AMPs, such as SIgA and lactoferrin, has been used as an indicator of URTI risk among athletes [31]. The SIgA acts on protecting the body as an immunological barrier from viral pathogens and signaling the presence of antigens to phagocytes through the mucosal surfaces [32]. In the present study, 4 weeks of vitamin D_3_ supplementation increased serum 25(OH)D concentration to sufficient levels, but it did not improve the SIgA concentration where both groups increased significantly from pre- to mid-tests. A previous study reported that the increase in SIgA concentration was observed after 4 weeks of intensified training in rhythmic gymnasts [33]. Another study supporting our results showed that while a significant increase in serum 25(OH)D concentration was observed from 54.5 to 125.5 nmol/L following 14 weeks of vitamin D_3_ supplementation (5000 IU/day), SIgA concentration was not different between the vitamin D and the placebo groups in healthy adults [25]. However, the author found that vitamin D_3_ supplementation facilitates SIgA secretion rate as well as salivary flow rate. Another study also reported that SIgA secretion rate was significantly higher in the optimal vitamin D group than in individuals with vitamin D insufficiency or deficiency [12]. They assumed that the increase in SIgA secretion rate plays a vital role in improving the resistance to respiratory infection. However, the present study did not measure the SIgA secretion rate and salivary flow rate that may limit comparison of the results with previous studies. 

Interestingly, the elevated salivary lactoferrin concentration in response to winter training was found only in the placebo group in the present study. Salivary lactoferrin is one of the AMPs that protect from respiratory infection, and it acts on the first line of defense against bacterial invasion from the environment [34]. He and colleagues reported that vitamin D status was not associated with AMPs concentrations such as lactoferrin, lysozyme, and SIgA [12], and correcting vitamin D level did not affect salivary lactoferrin concentration during the winter period [25]. It remains unclear why the lactoferrin level was increased only in the placebo group in the present study. It was assumed that the increase in lactoferrin concentration may be associated with susceptibility to respiratory infections. A greater salivary lactoferrin concentration has been observed following a single bout of cycling exercise [35] and oral patients with chronic periodontitis showed higher lactoferrin concentration than healthy adults [36].

### 4.2. Vitamin D_3_ Supplementation and URTI Symptoms

This study found that the vitamin D group was lower in URTI symptoms and total URTI symptoms than the placebo group, and the change in serum 25(OH)D concentration was negatively correlated with total URTI symptoms. Our findings were consistent with previous studies where vitamin D supplementation reduced the incidence of URTI infection or symptom scores [24,37,38]. Vitamin D_3_ supplementation with a dosage of 4000 IU/day reduced 23% of infection scores in patients who frequently had respiratory tract infections [37]. Another randomized and placebo-controlled study reported that a dose of 300 IU/day of vitamin D_3_ during the winter period (i.e., from January to March) reduced acute respiratory infections (odd ratio = 0.52) in vitamin D-deficient Mongolian children [38]. A recent individualized meta-analysis study also supports our results, reporting that vitamin D supplementation showed a 12% reduction in URTI [24].

As opposed to our results, three studies with athletes and young adults showed no effects of vitamin D supplementation on URTIs [17,39,40]. Dubnov-Raz and colleagues found that twelve weeks of vitamin D_3_ supplementation with a dosage of 2000 IU/day did not reduce the incidence and severity of URTI in adolescent swimmers [17]. Laaksi et al. [39] also found that 6 months of vitamin D_3_ supplementation did not change the number of days absent from military duty in young Finnish men. Monthly doses of 100,000 IU of vitamin D_3_ in healthy adults did not reduce the incidence or severity of URTIs in healthy adults [40]. However, there were interesting results in studies by Durbnov-Ras and Lakki et al. where decreased serum 25(OH)D concentration during winter season was negatively associated with the severity of URTIs in the placebo group [17], and the number of days absent from duty was lower among military individuals who decreased serum 25(OH)D concentration from 71.6 to 51.3 nmol/L during the winter season [39]. He and colleagues also pointed out that athletes with low serum 25(OH)D status (<30 nmol/L) were more likely predisposed to URTIs than athletes with greater than 120 nmol/L [12]. It is interesting that the benefits of vitamin D_3_ supplementation on URTI were not found in previous studies despite significant relations between vitamin D and URTI. Our finding was the first evidence of vitamin D_3_ supplementation providing a protective benefit against URTI symptoms among athletes. It was assumed that baseline vitamin D status might result in different outcomes between the previous and the present studies. In the previous studies, baseline of vitamin D status was greater than 70 nmol/L [17,39] while our subjects were 31.3 ± 1.39 nmol/L. Additionally, small changes in serum 25(OH)D concentration (16.9 nmol/L increase in swimmers and 2.l nmol/L decrease in young healthy adults vs. 71.4 nmol/L increase in taekwondo athletes) as well as low vitamin D_3_ dosage (2000 and 400 IU/day vs. 5000 IU/day) may affect the different results on URTIs. A recent meta-analysis study also supports our assumption that a great benefit of vitamin D supplementation on URTIs was found among individuals with low baseline 25(OH)D level (<25 nmol/L, odds ratio = 0.58) [24]. The author also described that daily or weekly vitamin D supplementation was more beneficial than a bolus dose to reduce the acute respiratory tract infection [24]. Other factors such as race, treatment periods, training intensity, and individual sensitivities to URTIs may influence the different outcomes. Nevertheless, our study confirmed that a daily dose of 5000 IU of vitamin D_3_ reduces URTI symptoms in vitamin D-insufficient taekwondo male athletes.

## 5. Limitation of Study

There are some limitations in the present study that should be considered when interpreting the results. Although the present study was designed systematically with a randomized controlled trial, small sample sizes may limit confirming the effectiveness of vitamin D_3_ supplementation on URTIs in athletes. An adequate number of subjects may be required in future studies to approach the clinical practice. Recruiting only male athletes also limits confirming the benefits of vitamin D_3_ supplementation in female athletes. Secondly, the Diasorin method can underestimate the serum 25(OH)D level compared to standardized assay such as LC-MS/MS [41,42]. However, this method has become the most popular assay in hospital and clinical laboratories, and it is an important tool for assessing an individual’s vitamin D level [43,44]. In addition, a previous study reported that the correlation between Diasorin and LC-MS/MS reflected more precisely at lower 25(OH)D concentration ranges [42]. We believe that Diasorin assay is suitable for the current study as we aimed to examine Vitamin D_3_ supplementation effects on URTI in the athletes who are considered as having Vitamin D insufficiency. Third, even though the present study used a smaller number of questions (11 questions) compared to other studies using questionnaires (i.e., 44 and 21 questions) [10,45] to increase the completion rate, the number of participants who completed the URTI questionnaire were only 17 subjects. It is assumed that lack of motivation with the questionnaire may decrease the returning rate despite the encouragement by coach and evaluators. A previous study with adolescent swimmers also reported low returning rate, indicating only 22 subjects (vitamin D group, *n* = 11, Placebo group, *n* = 11) completed the URTI questionnaire out of a total 55 subjects (vitamin D group, *n* = 28, placebo group, *n* = 27) [17]. Finally, the definition of URTI symptoms can be varied based on laboratory measurement, self-reported colds, and different questionnaires. Although clinically evaluated URTI episodes provide accurate information of true infections [46], the URTI symptoms evaluated by questionnaire should not be dismissed to identify the perceived conditions.

## 6. Conclusions

The present randomized controlled trial revealed that a daily dose of 5000 IU of vitamin D_3_ increases serum 25(OH)D concentration to a sufficient level, and the increased serum 25(OH)D level reduces the URTI symptoms following four weeks of winter training in vitamin D-insufficient male taekwondo athletes. Vitamin D_3_ supplementation may be effective in reducing the symptoms of URTI during winter training in vitamin D-insufficient taekwondo athletes.

## Figures and Tables

**Table 1:** Changes in body composition variables during 4 weeks of winter training.

Variables	Group	Pre	Post	*F*-value		
				G	T	G × T
Body weight (kg)	Vitamin D	78.6 ± 3.28	76.7 ± 2.98 ^+^	1.788	27.768 *	2.080
	Placebo	72.5 ± 2.84	71.5 ± 2.77 ^+^			
Lean body mass (kg)	Vitamin D	64.2 ± 2.12	64.1 ± 2.02	3.147	1.011	1.257
	Placebo	58.7 ± 1.98	59.3 ± 2.02			
Fat mass (kg)	Vitamin D	9.9 ± 1.19	9.3 ± 1.16 ^+^	0.086	6.916 *	0.000
	Placebo	9.5 ± 0.82	8.9 ± 0.67 ^+^			
BFP (%)	Vitamin D	12.7 ± 0.99	11.8 ± 0.93 ^+^	0.237	39.894 *	0.106
	Placebo	13.4 ± 0.67	12.3 ± 0.55 ^+^			

Note. BFP, body fat percentage; G, group; T, time; G × T, group by time; * *p* < 0.05 indicates significant time effects. ^+^ *p* < 0.05 indicates significant changes from pre- to post-tests within the group.

**Table 2:** The symptoms of URTI during winter training.

Variables	Vitamin D	Placebo	*U*-value	*p*-value
URTI symptom (score/day)	7.7 ± 1.06	13.0 ± 1.60	10.5	0.011
QOL (score/day)	7.0 ±1.00	9.7 ± 2.27	18.0	0.093
Total URTI symptom (score/day)	14.7 ± 1.64	22.7 ± 2.27	11.0	0.015
